# Low-Molecular-Weight Fe(III) Complexes for MRI Contrast Agents

**DOI:** 10.3390/molecules27144573

**Published:** 2022-07-18

**Authors:** Shangjun Chen, Lu An, Shiping Yang

**Affiliations:** 1 Key Laboratory of Resource Chemistry of Ministry of Education, Shanghai Key Laboratory of Rare Earth Functional Materials, and Department of Chemistry, Shanghai Normal University, Shanghai 200234, China; jshchen@shnu.edu.cn; 2 Shanghai Municipal Education Committee Key Laboratory of Molecular Imaging Probes and Sensors, Shanghai Normal University, Shanghai 200234, China; anlu1987@shnu.edu.cn

**Keywords:** Fe(III) complexes, MRI contrast agents, relaxivity, thermodynamic stability, structure relationship

## Abstract

Fe(III) complexes have again attracted much attention for application as MRI contrast agents in recent years due to their high thermodynamic stability, low long-term toxicity, and large relaxivity at a higher magnetic field. This mini-review covers the recent progress on low-molecular-weight Fe(III) complexes, which have been considered as one of the promising alternatives to clinically used Gd(III)-based contrast agents. Two kinds of complexes including mononuclear Fe(III) complexes and multinuclear Fe(III) complexes are summarized in sequence, with a specific highlight of the structural relationships between the complexes and their relaxivity and thermodynamic stability. In additional, the future perspectives for the design of low-molecular-weight Fe(III) complexes for MRI contrast agents are suggested.

## 1. Introduction

Contrast-enhanced magnetic resonance imaging (MRI) has become a valuable and established diagnostic imaging tool worldwide since the first contrast agent designed for MRI was approved for clinical use in 1988 [1,2,3]. Today, tens of millions of MRI tests are carried out for patients annually around the world. Among them, gadolinium(III) complex-based contrast agents are the most widely used agents; about 40% exams involve the use of gadolinium(III) complexes [4]. The advantages of gadolinium(III) complexes rely on their large magnetic susceptibility, strong nuclear relaxation, immediate imaging after injection, wide biodistribution, and fast pharmacokinetic clearance [5,6,7,8,9]. Gadolinium(III) complexes were considered among the safest contrast agents, however, in 2006, an association between the use of Gd(III)-based contrast agents (GBCA) and a pathological condition (nephrogenic systemic fibrosis, NSF) was identified in renally impaired patients [10,11]. Retention of Gd-containing species was also detected in the brain of patients after administration of Gd(III)-based contrast agents [12]. Since 2013, more clear evidences have been reported to support Gd(III) accumulation in the central nervous system after exposure to gadolinium [13,14,15]. Although the toxic effect related to Gd(III) retention has not yet been confirmed, the concerns about the long-term safety of Gd(III)-based agents have led to renewed interest in Fe(III) complexes as alternatives [16,17,18], which display a similar biodistribution and pharmacokinetic clearance to the clinically used Gd(III) agents. 

Relaxivity, one of the most fundamental and important properties of a contrast agent, is defined as the efficiency with which the complex enhances the relaxation rates of tissue water [1,2]. The relaxation rates of water are dependent on the longitudinal relaxation time (*T*_1_) or the transverse relaxation time (*T*_2_) of water. The extent to which a contrast agent can short the *T*_1_ or *T*_2_ of water is termed longitudinal relaxivity (*r*_1,_ defined as 1/*T*_1_) and transverse relaxivity (*r*_2_, defined as 1/*T*_2_), respectively. Contrast agents with a large *r*_1_ generally increase the signal and produce bright (*T*_1_-weighted) MR images, while contrast agents with a large *r*_2_ reduce the signal and make the MR images (*T*_2_-weighted) dark. The waters directly coordinating to the central metal ions (inner-sphere water), interacting with groups on the complex (second-sphere water), and the rapidly diffusing waters at the outer-sphere (outer-sphere water) can contribute to longitudinal relaxivity (*r*_1_) [1,2]. The inner- and outer-sphere mechanisms have been developed to describe the relaxation process of water molecules directly coordinated to the paramagnetic center and those present in the vicinity of the complex, respectively [19,20,21]. The inner-sphere contribution is represented by Equation (1), where *q* is the number of coordinated water molecules, *τ*_M_ is the residency lifetime of bound water, and *T*_1M_ is the relaxation time of bound water [1,22].

(1) $$r_{1}^{\mathrm{IS}}=\frac{q}{55.55}\frac{1}{T_{1M}+\tau_{M}}$$

The value of *T*_1M_ can be given by the Solomon–Bloembergen equations (Equation (2)) and Equation (3) at magnetic fields higher than 20 MHz. In Equation (2), γ_I_ is the proton gyromagnetic ratio, *g*_e_ is the electronic *g*-factor, *μ*_B_ is the Bohr magneton, *S* is the total electron spin of the metal ion, *r*_MH_ is the proton–metal ion distance, *τ*_c1_ is the correlation time, ω_I_ is the proton Larmor precession frequency. In Equation (3), *T*_1e_ is the electron spin relaxation and *τ*_R_ is the rotation dynamics of the complex [1,22]

(2) $$\frac{1}{T_{1M}}≅\frac{2}{15}\gamma_{I}^{2}g_{e}^{2}\mu_{B}^{2}S\left(S+1\right)r_{\mathrm{MH}}{}^{-6}\left[\frac{3\tau_{c1}}{1+\omega_{I}^{2}\tau_{c1}^{2}}\right]$$

(3) $$\frac{1}{\tau_{c1}}=\frac{1}{T_{1e}}+\frac{1}{\tau_{M}}+\frac{1}{\tau_{R}}$$

In spite of the relatively smaller spin quantum number for Fe(III) (*S* = 5/2) in comparison to Gd(III) (*S* = 7/2), Fe(III) complex-based contrast agents indeed show several advantages as follows [22,23,24]: (i) The Fe(III) ion is a very strong Lewis acid because of its small ionic radius and high charge. It can coordinate with suitable ligands to form robust complexes that are even more stable than their Gd(III)-based counterparts [25]. (ii) The relatively shorter metal ion-to-water hydrogen (*r*_MH_) distance in the Fe(III) complex can improve the *r*_1_ of the Fe(III) complex. (iii) The body has highly regulated systems to deal with the safe transport and storage for Fe^3+^ ions but not for Gd^3+^ ions. Hence, the toxicity of potentially released traces of Fe^3+^ ion could be lower than the Gd(III) ions for the long term [26]. (iv) It has been reported that the relaxivity of Fe(III) complexes increase with the enhancement of the magnetic field strength. However, the gadolinium-based counterparts generally show lower relaxivity at higher magnetic fields. This property makes Fe(III) complexes more desirable for modern MRI tomographs performed at higher magnetic fields [27,28]. 

In this mini-review, we focus on low-molecular-weight Fe(III) complexes designed for MRI contrast agents in the past ten years. We highlight the structure–property relationship between the chelates and the relaxivity and thermodynamic stability. We also describe their application for contrast enhancement in MRI. The Fe(III) complexes in this mini-review are simply divided into two categories including the mononuclear Fe(III) complexes and the multinuclear Fe(III) complexes. We summarize their recent progress in sequence and suggest our future perspectives on the development of Fe(III) complexes for applications in MRI. 

## 2. Mononuclear Fe(III) Complexes

### 2.1. Mononuclear Fe(III) Complexes with Open Ligands

Mononuclear Fe(III) complexes in this mini-review are divided into mononuclear Fe(III) complexes with open ligands and mononuclear Fe(III) complexes with macrocyclic ligands on the basis of the structural differences of the ligands. In this section, mononuclear Fe(III) complexes with open ligands are discussed. This kind of complex was the earliest and widest-studied contrast agent for MRI. One of the main advantages of linear Fe(III) complexes relies on the easily modification of the chemical structures of the ligands. Lots of Fe(III) complexes with or without coordinated water molecules were designed by employing ligands possessing different type of coordination groups such as amine, hydroxyl, pyridine, imidazole, and so on. 

**Figure 1:**
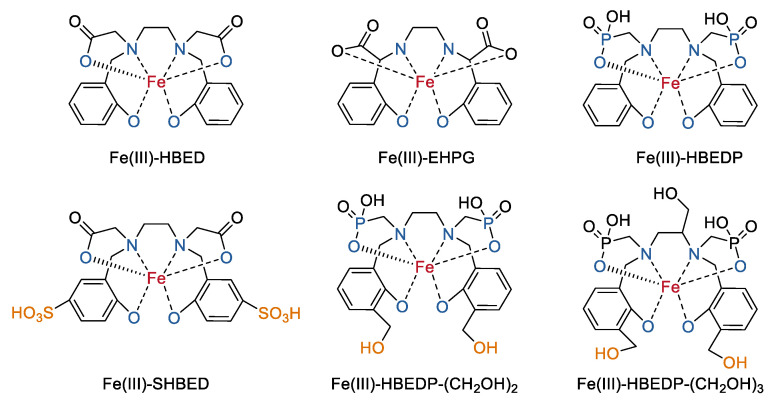
Chemical structures of mononuclear Fe(III) complexes with open ligands based on HBED and its derivatives [33].

Ligands containing ethylene diamine backbones with phenol and carboxylate pendants were among the earliest studied ligands for mononuclear Fe(III) complexes with open ligands [29,30,31]. These ligands such as N, N’-bis (2-hydroxyphenyl) ethylenediamine-N, N’-diacetic acid (HBED) and ethylenebis (2-hydroxypheny1) glycine (EHPG) bind tightly to Fe^3+^ ions to form six-coordinate stable Fe(III) complexes (Figure 1). Due to the phenyl unit in the ligands, these complexes (for example, Fe(III)-HBED and Fe(III)-EHPG) can also bind strongly to human serum albumin and have been explored as liver-specific MRI contrast agents in preclinical studies [32]. In contrast, in order to construct stable extracellular fluid (ECF) contrast agents with improved contrast and lower serum protein binding relative to HBED-based complexes, Bruce F. Johnson and co-workers recently developed more hydrophilic analogs by adding hydroxyl groups and/or charge to HBED [33]. The acetic acid group in HBED was replaced by phosphoric acid (HBEDP), an additional sulfonate group was introduced into the phenyl unit in HBED (SHBED), and an additional two or three hydroxyl groups were further introduced into HBEDP (HBEDP-(CH_2_OH)_2_ and HBEDP-(CH_2_OH)_3_) (Figure 1). The *r*_1_ relaxivity of these analog complexes (Fe(III)–HBEDP, Fe(III)–SHBED, Fe(III)–HBEDP–(CH_2_OH)_2_, and Fe(III)–HBEDP–(CH_2_OH)_3_) were all increased by two to three times in comparison to that of Fe(III)–HBED (Table 1). Increased second sphere hydration could be one of the possible explanations because of the improved interaction between the hydrophilic sites in these complexes and water. The ratio between the *r*_2_ and *r*_1_ values for these complexes were even comparable to clinically approved GBCA at 1.5 T in water [28]. The largest decrease of serum protein binding was observed in Fe(III)–SHBED, which showed no appreciable obvious serum protein binding. The binding capability of Fe(III)–HBEDP with a greater negative charge was increased slightly, while the binding capability of analogs with additional hydroxyl groups was reduced relative to Fe(III)–HBED. MRI of a mouse tumor model indicated that Fe(III)–SHBED, Fe(III)–HBEDP–(CH_2_OH)_2_, and Fe(III)–HBEDP–(CH_2_OH)_3_ have relative lower liver signal enhancement levels in comparison to that of Fe(III)–HBED.

**Figure 2:**
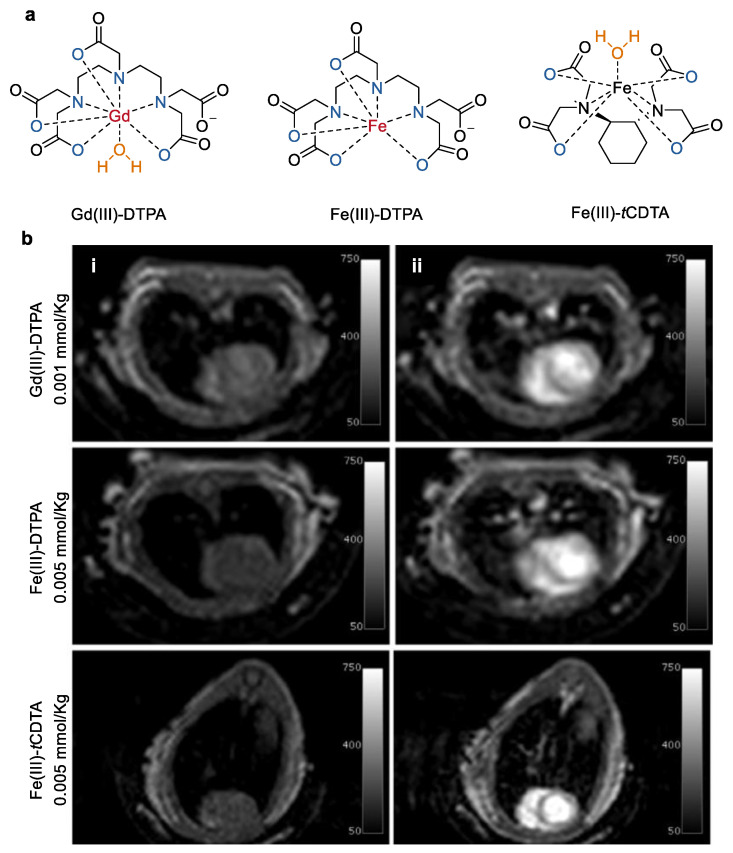
(**a**) Chemical structures of mononuclear Fe(III) complexes with open ligands based on DTPA and *t*CDTA, the reference Gd(III)–DTPA complex was also shown, and (**b**) *T*1 contrast MRI of mouse hearts before (**i**) and after injection (**ii**) of Gd(III)–DTPA, Fe(III)–DTPA, and Fe(III)–*t*CDTA at 1.5 T [25].

Fe(III) complexes even show higher stability than Gd(III) complexes. For example, as reported by Eyk A. Schellenberger and co-workers [25], the log *K*_ML_ for iron chelates of pentetic acid (Figure 2, Fe(III)–DTPA, log *K*_ML_ = 27.3) and of *trans*-cyclohexane diamine tetraacetic acid (Fe(III)–*t*CDTA, log *K*_ML_ = 27.5) was about five orders of magnitude higher than that of Gd(III)–DTPA (log *K*_ML_ = 22.5, Table 1) [34]. Both Fe(III)–DTPA and Fe(III)–*t*CDTA have seven coordination sites of Fe^3+^, but the Fe^3+^ in Fe(III)–*t*CDTA leaves one side available for water coordination. As a result, the relaxivity in serum of Fe(III)–*t*CDTA (*r*_1_ = 2.2 mmol^−^^1^ s^−^^1^, *r*_2_ = 2.5 mmol^−^^1^ s^−^^1^) measured at 0.94 T at room temperature was approximately threefold higher compared with those of Fe(III)–DTPA (*r*_1_ = 0.9 mmol^−^^1^ s^−^^1^, *r*_2_ = 0.9 mmol^−^^1^ s^−^^1^). It should be mentioned that these relaxivities of the iron chelates were relatively lower than that of Gd(III)–DTPA (*r*_1_ = 4.1 mmol^−^^1^ s^−^^1^, *r*_2_ = 4.8 mmol^−^^1^ s^−^^1^), where the coordination sides of Gd^3+^ were occupied by the DTPA chelator and one exchangeable water molecule. However, *T*1-weighted MR imaging of a breast cancer xenograft mouse model indicates that the Fe(III) complex used at a higher concentration (at 0.5 mmol/kg for Fe(III)–DTPA and 0.2 mmol/kg for Fe(III)–*t*CDTA) shows a comparable *T*1 effect to that of Gd(III)–DTPA (at 0.1 mmol/kg). Moreover, both Fe(III)–DTPA and Fe(III)–*t*CDTA have similar volume transfer constant values to that observed for Gd(III)–DTPA under the same measurement conditions. Based on these findings, the authors proposed that the low-molecular-weight Fe(III) complexes may be a promising alternative contrast agent for *T*1-weighted MR imaging with the concern about the long-term safety of Gd(III) complex. 

**Figure 3:**
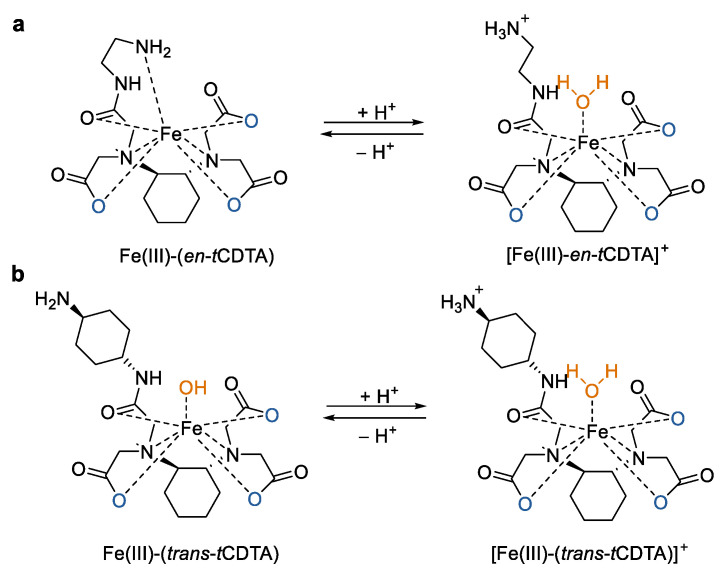
Chemical structures of mononuclear Fe(III) complexes with open ligands and their mechanisms of the observed pH-dependent relaxivity changes of the complexes. (**a**) Fe(III) complexes based on *en*-*t*CDTA and (**b**) Fe(III) complexes based on *trans*-*t*CDTA [35].

In order to further improve the properties of *t*CDTA-based Fe(III) complexes, they altered the structure of the *t*CDTA ligand by coupling amine-containing compounds (ethylenediamine (*en*) and *trans*-1,4-diaminocyclohexane) to one carboxyl group of it (Figure 3) [35]. Four *t*CDTA derivatives including monomers (*en*-*t*CDTA and *trans*-1,4-diaminocyclohexane–*t*CDTA) and dimers (*en*-*t*CDTA-dimer and *trans*-1,4-diaminocyclohexane–*t*CDTA-dimer) and their complexes with Fe^3+^ ions were prepared. All of the four Fe(III) complexes displayed high stability as Fe(III)–*t*CDTA. However, the relaxivity of the ethylenediamine derivatives-based complexes (Fe(III)–(*en*-*t*CDTA) and Fe(III)–(*en-*Di-*t*CDTA)) were significantly reduced in comparison to that of Fe(III)–*t*CDTA at 0.94 T and neutral pH. This is more likely due to the coordination of the terminal amine group of the ligands instead of a water molecule with the central Fe^3+^ ion, thereby preventing the inner-sphere relaxation. The strong pH dependence of relaxivity for these complexes with a substantial increase from neutral pH toward pH 5.0 supported the above conclusion and also made them potential candidates for MRI contrast agents of tumors [36]. In the case of diaminocyclohexane derivative-based complexes (Fe(III)–(*trans*-*t*CDTA) and Fe(III)–(*trans*-Di-*t*CDTA)), they showed comparable relaxivities to Fe(III)–*t*CDTA at 0.94 T and a neutral pH. However, the relaxivity decrease at pH above 7.4 was mainly attributable to the deprotonation of the coordinated water molecule. Moreover, the *r*_1_ relaxivities of these complexes increased with higher magnetic field strengths from 1.5 T to 7.0 T [37].

**Figure 4:**
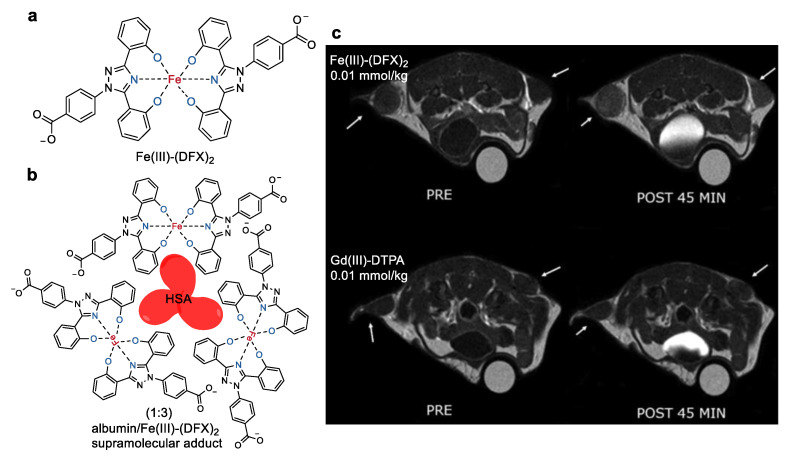
(**a**) Chemical structures of Fe(III)–(DFX)_2_, (**b**) albumin/Fe(III)–(DFX)_2_ supramolecular adduct, and (**c**) *T*1-weighted images of the TS/A-inoculated mouse with or without administration of Fe(III)–(DFX)_2_ and Gd(III)–DTPA [39]. The arrows indicate the tumor regions. Reprinted/adapted with permission from Ref. [39]. Copyright 2021 American Chemical Society.

Deferasirox (DFX), commonly used iron sequestering agents in thalassemic patients [38], was chosen as a biocompatible ligand by the group of Eliana Gianolio to chelate the iron ions, leading to the formation of a hexacoordinated Fe(III) complex with two units of the ligand (Figure 4) [39]. The complex showed extremely high thermodynamic stability (log *K_ML_* = 38.6 [40]) and a favorable clearance via the hepatobiliary [41]. Since DFX can tightly bind to human serum albumin (HSA), its complex also displayed a high binding affinity to HSA. Consequently, an obvious increase in both longitudinal (*r*_1_) and transverse (*r*_2_) relaxivities in human serum was observed at 0.47 T and 1 T, and at pH 7.4 (Table 1). The variable temperature of the ^17^O-*T*_2_-NMR experiment suggested the absence of any inner sphere water molecules coordinated to the Fe(III) ion [42]. The large relaxivities in human serum were then attributed to the water molecules in the second coordination sphere. The binding capacity of the complex to HSA was studied in detail by the titration experiments with different concentration of the complex or HSA [43]. The results indicated that three Fe(III)–(DFX)_2_ units bind tightly to one molecule of HSA with an average apparent binding constant (*K*a) of 2.8 × 10^5^ M^−1^. The specific binding sites on HSA were also determined by supplemental titrations in the presence of competitive drugs (ibuprofen, iodipamide, and methyl orange), which can selectively occupy the subdomains IB, IIA (Sudlow site I), and IIIA (Sudlow site II) of HSA. The findings proved the strongest binding sites for Fe(III)–(DFX)_2_ on albumin were the IB and IIA sites. *T*_1_-weighted MRI images on a tumor-bearing mouse on a 3 T MRI scanner indicate that Fe(III)–(DFX)_2_ produced a comparable imaging contrast to that of the commercial Gd(III)–DTPA agent (Figure 4c).

**Figure 5:**
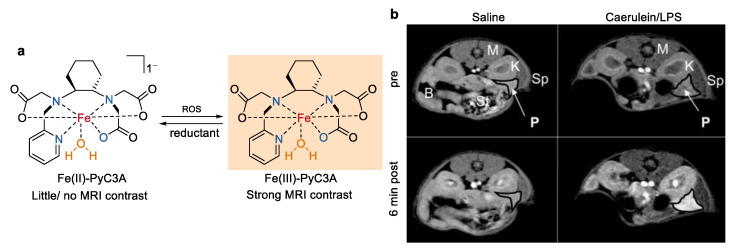
(**a**) Chemical structures of Fe(II)–PyC3A, Fe(III)–PyC3A, and reversible transformation between them with oxidation and reduction. ROS represent the reactive oxygen species. (**b**) *T*1-weighted images of saline and caerulein/LPS-treated mice recorded before and after injection of 0.2 mmol/kg Fe(II)–PyC3A [48]. Organs in Figure (**b**) are labeled as follows: bowel (B), kidney (K), muscle (M), pancreas (P), Sp (spleen), and St (stomach). Reprinted/adapted with permission from Ref. [48]. Copyright 2019 American Chemical Society.

Biochemically responsive MRI contrast agents that provide positive signal enhancement in the presence of a specific biochemical target have received widely attentions in recent years [44,45,46]. Redox-active iron complexes with different metal oxidation states are among the most attractive candidates [47]. As shown in Figure 5, a redox-active Fe complex (Fe-PyC3A) that responds to reactive oxygen species (ROS) was reported by Eric M. Gale and co-worker recently for the MR imaging of pathologic changes in vivo [48]. On the basis of their previous study of a Mn(II) complex [49], a ligand possessing amino and carboxylate groups (PyC3A) was used to chelate both the Fe^3+^ and Fe^2+^ ions. Large stability constants were determined for Fe(III)–PyC3A (Log *K*_ML_ = 23.2 ± 1.8) and Fe(II)–PyC3A (Log *K*_ML_ = 15 ± 1.8), indicating that PyC3A can supports stable complexes of Fe^3+^ and Fe^2+^. As expected, the *r*_1_ relaxivity of Fe(III)–PyC3A was an order of magnitude higher than that of Fe(II)–PyC3A because of the distinct paramagnetic properties of these two complexes. *T*_1_-weighted images in mice in vivo also indicated that Fe(III)–PyC3A generated substantial stronger signal enhancement than that of Fe(II)–PyC3A. Fe^3+/2+^–PyC3A has a redox potential of 230 mV versus NHE. Fe(III)–PyC3A was ready reduced into Fe(II)–PyC3A by L-cysteine (*E*^1/2^ cited between −150 and 250 mV vs NHE [50]), while Fe(II)–PyC3A was rapidly oxidized back to the corresponding Fe^3+^ complex by H_2_O_2_ (*E*^red^ = 0.38 V vs NHE [51]). Fe–PyC3A was then used to detect ROS production in a murine model of acute pancreatitis. Only strong and selective signal enhancement was observed in the caerulein/LPS-treated mice with an inflamed pancreas after injection of Fe^2+^–PyC3A. It’s worth noting that this study was the first example of using metal ion redox to visualize pathologic changes in vivo.

**Table 1:** Formation constants (log *K*_ML_), relaxivity (*r*_1_, *r*_2_), and hydration state (*q*) for mononuclear Fe(III) complexes with open ligands.

Complex	Log *K*_ML_	*r*_1__(mM_^−1^_s_^−1^_)_ in Buffer	*r*_2__(mM_^−1^_s_^−1^_)_ in Buffer	*q*
Fe(III)–HBED [31]	39.7	0.49 ^a^	0.52 ^a^	0
Fe(III)–HBEDP [33]	-	0.9 ^a^	1.0 ^a^	0
Fe(III)–SHBED [33]	-	1.3 ^a^	1.6 ^a^	
Fe(III)–HBEDP- (CH_2_OH)_2_ [33]	-	0.9 ^a^	1.2 ^a^	0
Fe(III)–HBEDP- (CH_2_OH)_3_ [33]	-	1.5 ^a^	1.7 ^a^	0
Gd(III)–DTPA [25]	22.5	3.4 ^b^	3.9 ^b^	1
Fe(III)–DTPA [25]	27.3	0.6 ^b^	0.6 ^b^	0
Fe(III)–*t*CDTA [25]	27.5	2.0 ^b^	2.2 ^b^	1
Fe(III)–*t*CDTA [35]	27.5	1.56 ^c^	1.72 ^c^	1
Fe(III)–(*en*-*t*CDTA) [35]	-	0.78 ^c^	0.81 ^c^	0
Fe(III)–(*trans*-*t*CDTA) [35]	-	1.92 ^c^	2.16 ^c^	1
Fe(III)–(DFX)_2_ [39]	38.6	2.3 ^d^	3.1 ^d^	0
Fe(III)–PyC3A [48]	23.2 ^e^	1.8 ^f^	-	1

^a^ Relaxivity values measured in phosphate-buffered saline at 1.4 T and 40 °C, ^b^ Relaxivity values measured in water, ^c^ Relaxivity values measured in water, at 0.94 T, pH 7.4, and 37 °C, ^d^ Data measured in water and in human serum at 1 T, pH 7.4, and 25 °C. ^e^ log *K*_ML_ vs free PyC3A under equilibrium conditions at pH 7.4. ^f^ Relaxivity values measured in Tris Buffer at 1.4 T, pH 7.4, and 37 °C.

### 2.2. Mononuclear Fe(III) Complexes with Macrocyclic Ligands

Mononuclear Fe(III) complexes with macrocyclic ligands with remarkable aqueous solubility, kinetic inertness to dissociation under biological environments, and tunable spin and oxidation states have attracted much attention over the past decades. Macrocyclic coordination ligands that enable exquisite control of the above mentioned properties of the Fe(III) complexes have developed by modifying the cavities of the macrocycles and/or the pendant groups on the macrocycles.

**Figure 6:**
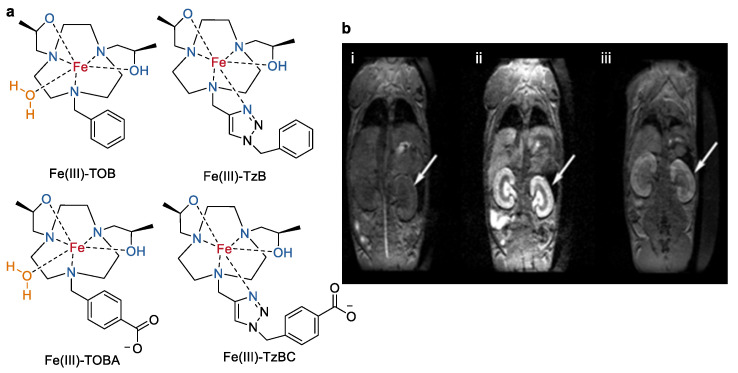
(**a**) Chemical structures of mononuclear Fe(III) complexes of Fe(III)–TOB, Fe(III)–TOBA, Fe(III)–TzB, and Fe(III)–TzBC. (**b**) *T*1-weighted MRI images of a healthy Balb/C mouse before and after injection of 0.05 mmol/kg Fe(III)–TOB at 4.7 T. Enhancement of kidneys (arrow) (**i**) before, (**ii**) 45 min, and (**iii**) 4 h post-injection [54]. Reprinted/adapted with permission from Ref. [54]. Copyright 2019 Wiley VCH.

Much attention have been devoted by the group of Janet R Morrow for the development of Fe(III) macrocyclic complexes based on the early studied triazacyclononane (TACN) macrocycles [52,53]. As shown in Figure 6, TACN with two pendent chiral hydroxyl–propyl groups and a third benzyl group were designed and used as the preferred ligand framework to produce Fe(III) complexes (Fe(III)–TOB and Fe(III)–TOBA) with a coordination site for a water ligand [54]. Reference coordinative saturated Fe(III) complexes (Fe(III)–TzB and Fe(III)–TzBC) based on TACN macrocycles containing the same alcohol groups and one additional triazole group were also prepared (Figure 6). These complexes were inert to dissociation in acid solution (100 mM HCl) for 4 h or in solutions with 25 mM carbonate and 0.40 mM phosphate for 72 h at 37 °C. Variable temperature ^17^O NMR spectroscopy experiments indicated that there was no inner-sphere water exchanging occurring sufficiently rapidly on the NMR time scale for these complexes. However, Fe(III)–TOB and Fe(III)–TOBA generated a higher relaxivity (*r*_1_ and *r*_2_) than that of Fe(III)–TzB and Fe(III)–TzBC both in buffer and human serum albumin (HSA) at 4.7 T, pH 7.2, and 37 °C (Table 2). Impressively, the *r*_1_ value of Fe(III)–TOB was comparable and the *r*_2_ value was even larger than that of the approved clinically used agent Gd(III)–DTPA. Dynamic MRI studies in mice at 4.7 T at a dose of 0.05 mmol/kg of Fe(III)–TOB showed a better and specific kidney contrast enhancement at 30 min post injection in comparison to Gd(III)–DTPA. This study represented an excellent example of structure–activity investment on Fe(III) complexes towards their further application as MRI contrast agents. 

**Figure 7:**
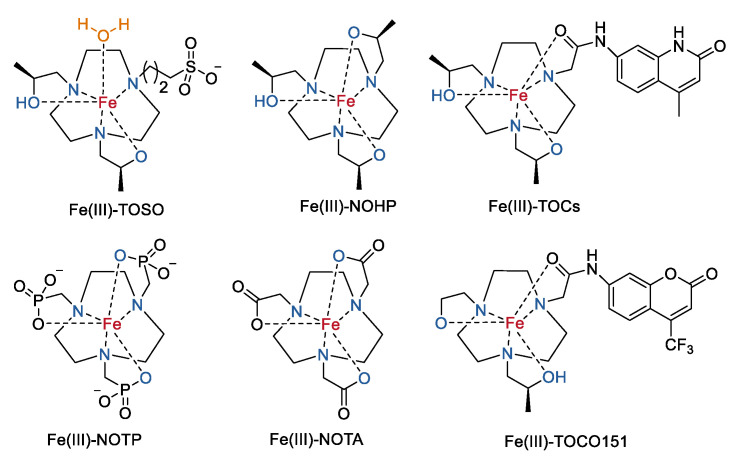
Chemical structures of mononuclear Fe(III) complexes based on TOB derivatives [55,56,57].

They further studied the effect of the third ancillary groups on the solution chemistry and relaxivity for the TACN macrocycle-based analogue in Fe(III) complexes (Figure 7). For the Fe(III) complexes with an inner-sphere coordinated water ligand, the replacement of the benzyl groups with an anionic non-coordinating sulfonate group (Fe(III)–TOSO) increases the aqueous solubility and the transverse relaxivity (*r*_2_)_,_ while maintaining the stability and the longitudinal relaxivity (*r*_1_) of the complex [55]. The better binding capacity of Fe(III)–TOSO to blood proteins such as serum albumin in comparison to other complexes may be part of the reasons. Generally, changing the third ancillary group into a coordination group such as the above triazole groups or the hydroxypropyl (Fe(III)–NOHP) [55], phosphonate (Fe(III)–NOTP) [56], carboxylate (Fe(III)–NOTA) [56], or amides (Fe(III)–TOCs or Fe(III)–TOCO151) [57] pendants produces coordinatively saturated Fe(III) complexes without the inner-sphere water ligands, and therefore giving rise to lower relaxivities of the Fe(III) complexes (Table 2). 

**Figure 8:**
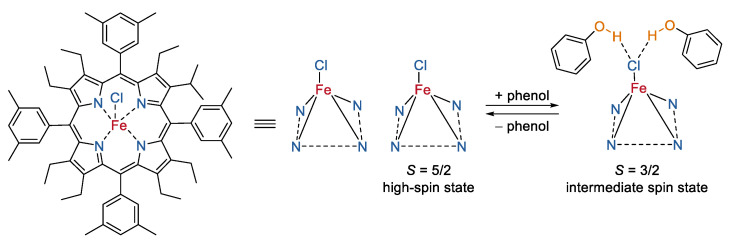
A spin-flip in Fe(III) porphyrin complexes responding to the presence of an H bonding partner [59].

Fe(III) macrocyclic complexes with switching spin-states upon stimulus have significant potential to provide molecular MRI contrast agents that respond by a change in their relaxivity [58]. One of example is the hydrogen bonding interaction-triggered spin-state switching of a five-coordinate iron-(III) octaethyltetraarylporphyrin chloride reported by de Visser and Prasad Rath [59]. As shown in Figure 8, the addition of phenol into the solution of iron(III) porphyrin produced a new Fe(III) complex with hydrogen-bonding interactions between the axial chloride and the proton of the phenol. As a result, the high-spin state (*S* = 5/2) of iron was switched into an intermediate spin state (*S* = 3/2), which was comprehensively proven by Mössbauer spectra, proton NMR spectra, and density functional theory calculations. This study provided a beautiful example of weak external perturbations to switch the spin states of Fe(III) macrocyclic complexes, but the experiments were conducted in organic solution instead of aqueous solution, and the relaxivities of these two states were not reported.

**Figure 9:**
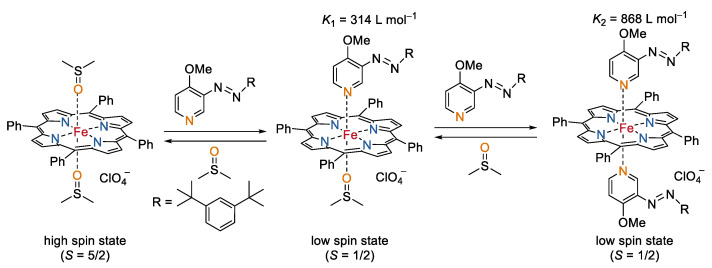
Switching of the spin state of Fe(III) porphyrin with the photo-switching ligand azopyridine [62].

The spin states of Fe(III) can also be switched by the stimulation of light. In their continuing studies on light-driven coordination induced spin-state-switches [60,61], the group of Herges recently reported an interesting light-controlled molecular spin switch based on a Fe(III) porphyrin (Figure 9) [62]. The Fe(III) porphyrin formed a high-spin (*S* = 5/2) complex with two axial DMSO ligands in a mixed acetone/DMSO solution (DMSO–acetone, 2:598). Impressively, in the presence of a photo-switchable azopyridine, the spin states between the high-spin (*S* = 5/2) and low-spin (*S* = 1/2) of the complex in solution (DMSO-acetone, 2:598) can be reversibly switched by changing the coordination geometry of the complex. The *trans* azopyridine with a suitable coordination structure to the Fe(III) porphyrin showed a binding constant 180 times higher than that of the DMSO ligands. Consequently, the two axial DMSO ligands can be substituted by two *trans* azopyridine ligands, leading to a low spin (*S* = 1/2) complex. In contrast, the *cis* isomer does not bind to the iron porphyrin due to the sterical hindrance. It can be replaced by DMSO, and therefore regenerating the high-spin state. Since the azopyridine can be reversibly changed between its *trans* and *cis* isomers by alternative irradiation with UV (365 nm) and visible light (450 nm), the ligand exchange/spin switching process was reversible. Notably, the switching cycles can be performed more than 1000 times under air and moisture at room temperature without any observed fatigue or side reactions. The switching of the spin states induced an outstanding change in the nuclear spin relaxation of the complex. The *r*_1_ relaxivity of the high spin complex was reported to be 17.7 times larger than that of the low spin complex in mixed acetone-*d*_6_/DMSO-*d*_6_ solution containing 1% water.

It is worth noting that there are also novel Fe(II) macrocyclic complexes with switching spin-states. For example, Jens Hasserodt and co-workers have developed several interesting irreversible spin-state-switching Fe(II) complexes that comprise the macrocycle TACN. The structural modification of these systems induced by a chemical reaction leaded to an irreversible change of the Fe(II) ions from low-spin state (*S* = 0) into the high-spin state (*S* = 1/2). According, a remarkable increase of the relaxivity can be obtained [63,64,65].

## 3. Multinuclear Fe(III) Complexes

Multinuclear Fe(III) complexes with more than one iron nuclears are generally more effective toward the development of iron-based MRI contrast agents. The relatively large size and the rigid geometry of the multinuclear complexes could slow localized molecular rotational motions. As a result, improved proton relaxivity can be expected. The strategy to create multinuclear complexes is to link two or more Fe(III) centers together. Rigid aromatic building blocks or *μ*-oxo bridge are the most used linkers. 

**Figure 10:**
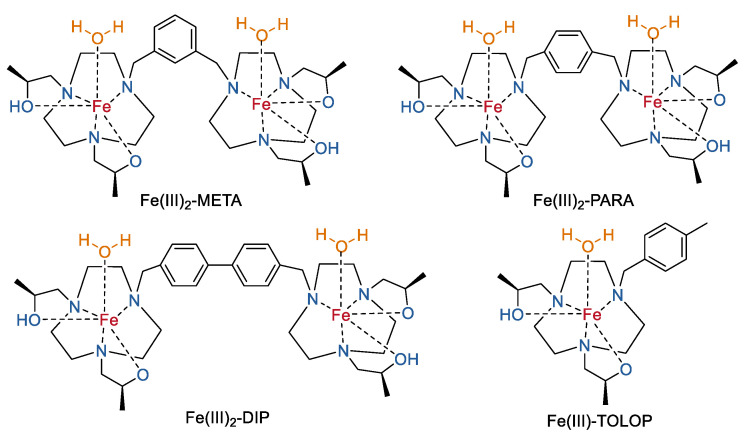
Chemical structures of dinuclear Fe(III) complexes based on TACN. A reference mononuclear complex (Fe(III)–TOLOP) was also shown [66].

Based on their studies of the TACN-containing Fe(III) macrocyclic complexes, Janet R Morrow and co-workers prepared several multinuclear Fe(III) complexes by linking two TACN-coordinated Fe(III) macrocyclic complexes together with an aryl (Fe(III)_2_-META and Fe(III)_2_-PARA) or biphenyl group (Fe(III)_2_-DIP) (Figure 10) [66]. These complexes maintain good aqueous solubility and robust kinetic inertness as the mononuclear complex (Fe(III)–TOB) [54]. Variable-temperature ^17^O NMR studies confirmed the lack of a rapidly exchanging inner-sphere water molecule of the dinuclear complexes [67], which is also similar to the mononuclear complex (Fe(III)–TOB). In contrast, the dinuclear Fe(III) complexes shown two times larger *r*_1_ relaxivity than that of the mononuclear complex at field strengths ranging from 1.4 T to 9.4 T in buffer (Table 3). The *r*_1_ relaxivity of Fe(III)–PARA was even almost 3-fold larger than that of the mononuclear complex of Fe(III)–TOLOP at 4.7 T. Limited rotational motions of the dinuclear complexes with larger molecular weights may partly contribute to the large *r*_1_ relaxivity. Due to the higher cationic charge on these dinuclear complexes, they showed increased lipophilicity in comparison to the mononuclear complexes. Accordingly, better binding capacities to serum albumin (4.5% *w*/*v*) at pH 7.2, 37 °C were observed and therefore a 1.2- to 1.6-fold increase of the *r*_1_ values in HSA were recorded for the dinuclear iron complexes.

**Figure 11:**
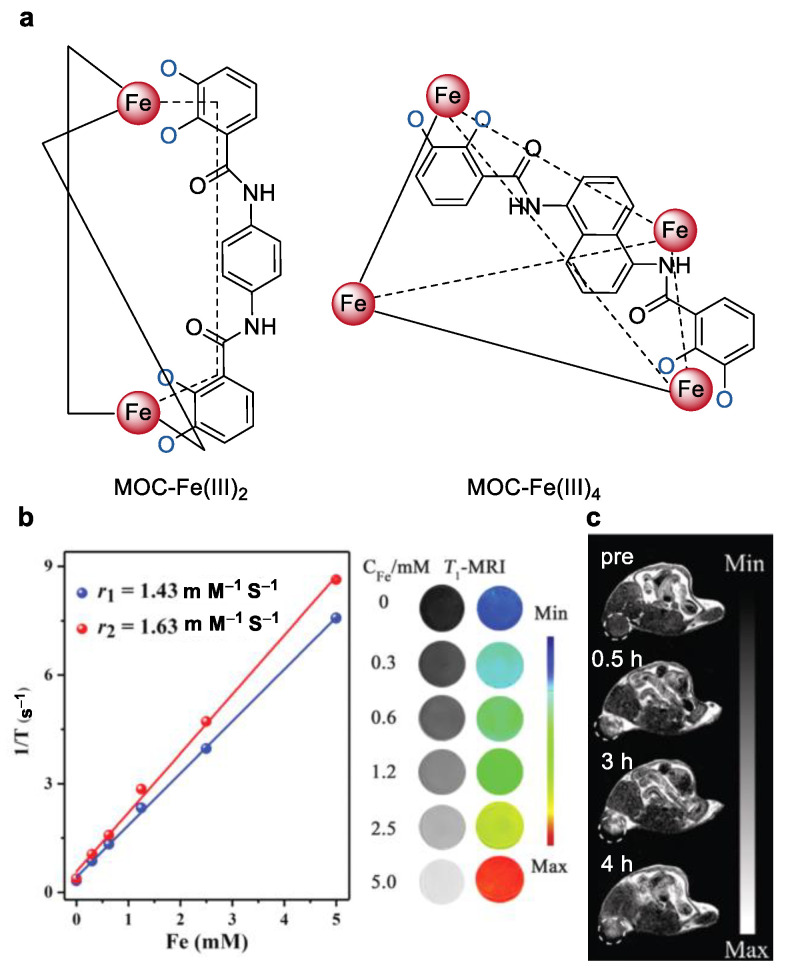
(**a**) Chemical structures of the metal–organic cages with two (MOC–Fe(III)_2_) and four Fe(III) centers (MOC–Fe(III)_4_), (**b**) relaxivities of MOC–Fe(III)_4_ in solution and the corresponding MRI images, and (**c**) in vivo magnetic resonance images of BALB/c mice with 4T1 tumors before and after intratumor injection of MOC–Fe(III)_4._ [68]. Reprinted/adapted with permission from Ref. [68]. Copyright 2019, Royal Society of Chemistry.

The recently independent studies from our group [68,69] and the group of Janet R Morrow [70] demonstrated that water soluble metal–organic cages (MOC) with multiple Fe(III) centers are promising alternatives to prepare multinuclear Fe(III) complexes [71]. In our study, two water soluble metal-organic cages with two (MOC-Fe(III)_2_) and four Fe(III) centers (MOC–Fe(III)_4_) based on the bisbidentate catecholate ligand were prepared (Figure 11) [68]. The dinuclear triple helicate configuration of MOC-Fe(III)_2_ as well as the tetrahedral configuration of MOC–Fe(III)_4_ was confirmed by the quadrupole time-of-flight mass spectrometry (Q-TOF-MS), UV-Vis spectroscopy, and IR spectroscopy. The hydrophilic catecholate ligands endow the good aqueous solubility of the complexes, which dissolve completely in water. The longitudinal (*r*_1_) and transversal (*r*_2_) relaxivity values in solution were determined to be 1.07, 1.43 mM^−1^ s^−1^ and 1.28, 1.63 mM^−1^ s^−1^ for complexes MOC–Fe(III)_2_ and MOC–Fe(III)_3_, respectively. A clear increase of the relaxivity per molecule could be observed for complex the MOC–Fe(III)_4_ with four Fe(III) centers. The relatively larger bulky structure of MOC–Fe(III)_4_ (overall size was 19.3 × 19.3 × 19.3 Å^3^) in comparison to that of MOC–Fe(III)_2_ (overall size was 18.6 × 13.7 × 13.7 Å^3^) may slow down the molecule rotation and therefore increase the rotational correlation time. This could be one of the reasons of the increased relaxivity for MOC–Fe(III)_4_ [72]. MTT studies with HUVEC, 4T1 and BT474 cells after incubation with the complex MOC–Fe(III)_4_ for 12 h were shown to exceed 80% cell viabilities, suggesting the good biocompatibility of the complexes. In vivo MRI imaging studies of BALB/c mice with 4T1 tumors after injection with MOC–Fe(III)_4_ showed an obvious contrast enhancement in the tumor region.

**Table 3:** Relaxivity (*r*_1_, *r*_2_) for multinuclear Fe(III) complexes.

Complex	*r*_1 (mM_^−1^_s_^−1^_)_^a^ in Buffer	*r*_2 (mM_^−1^_s_^−1^_)_^a^ in Buffer	*r*_1 (mM_^−1^_s_^−1^_)_ in HSA	*r*_2 (mM_^−1^_s_^−1^_)_ in HSA
Fe(III)–TOLOP [66]	1.83	5.81	2.71	4.32
Fe(III)_2_-META [66]	4.06	13.70	6.56	8.85
Fe(III)_2_-PARA [66]	5.26	13.44	6.71	11.90
Fe(III)_2_-DIP [66]	4.36	10.93	5.82	7.56
MOC-Fe(III)_2_ [68]	1.07 ^b^	1.28 ^b^	-	-
MOC-Fe(III)_4_ [68]	1.43 ^b^	1.63 ^b^	-	-
MOC-Fe(III)_4_ [70]	8.7 ^c^	-	21 ^c^	-

^a^ Relaxivity values measured in 100 mM NaCl, 20 mM HEPES buffer, at 4.7 T, pH 7.2 and 37 °C. pH 7.2,. ^b^ Relaxivity values measured in solution at 1.0 T. ^c^ Relaxivity values measured in phosphate buffer saline at 4.7 T, pH 7.4 and 37 °C.

The relaxation properties of the metal–organic cage MOC–Fe(III)_4_ also piqued the interest of the Morrow group [70]. The cage showed remarkable kinetically inertness in the presence of excess of other competitive binding ions or ligands, such as phosphate anions, Zn(II) cations, or ethylenediaminetetraacetic acid (EDTA). In contrast, the simple catechol complexes of iron rapidly decompose in the presence of Zn(II) cations or EDTA.

**Figure 12:**
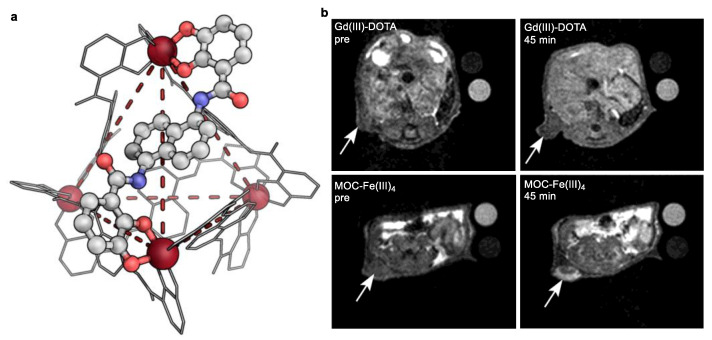
(**a**) SC-XRD model of MOC–Fe(III)_4_ superimposed over a tetrahedron (red dashes). H atoms, counter-ions, and solvent were removed for clarity, (**b**) in vivo magnetic resonance images of subcutaneous ID8 ovarian cancer tumors before and after injection of Gd(III)–DOTA (50 μmol/kg) or MOC–Fe(III)_4_ (12.5 μmol/kg). The arrows indicate the tumor regions [70]. Reprinted/adapted with permission from Ref. [70]. Copyright 2022 American Chemical Society.

EPR spectroscopy with a prominent feature at *g* ≈ 4.23 supported the characteristic Fe(III) high-spin center in a triscatecholate environment of the cage [73]. Variable-temperature ^17^O NMR spectroscopy experiments also confirmed the coordinative saturated Fe center without any inner-sphere water ligands. MOC-Fe(III)_4_ produced a good *r*_1_ relaxivity of 8.7 mM^−1^ s^−1^ per molecule or 2.2 ± 0.1 mM^−1^ s^−1^ per iron at 4.7 T and 37 °C in buffer at pH 7.4 (Table 3). This relaxivity was comparable to the relaxivity of the macrocyclic counterparts Fe(III)–TOB. MOC–Fe(III)_4_ displayed a marked three-fold increases in relaxivity (21 mM^−1^ s^−1^) in the presence of HSA, which was due to the strong binding interaction between the cage and HSA (*K*a ≈ 10^5^ M^−1^). Accordingly, the metal–organic cage showed promising comparable tumor enhancement properties relative to Gd(III)–DOTA in the vascular system in BALB/c mice (Figure 12).

## 4. Conclusions and Outlook

The development of iron complexes as contrast agents for MRI has again attracted much attention due to the long-term safety concerns about Gd(III)-based agents in recent years. Fe(III) complexes have been considered as one of the promising alternatives because of their high thermodynamic stability, relatively low long-term toxicity, and large relaxivity at higher magnetic field. This mini-review has attempted to provide an overview of recent progress of low-molecular-weight Fe(III) complexes designed for MRI contrast agents and summarize the strategies to modify their relaxivity and thermodynamic stability. Changing the coordination groups and/or introducing suitable hydrophilic groups of the previously studied chelators (for example, HBED, EHPG, and TACN) can largely alter the coordination structures of the central iron ions (with or without inner-sphere water ligand), the second coordination sphere environment, as well as the charge of the complexes, which gives rise to the enhance of relaxivities and the improvement of thermodynamic stability. Clinically used iron-sequestering agents with a high biocompatibility and strong binding ability to Fe^3+^ could be also a good choice of chelators for Fe(III) complexes. Multinuclear Fe(III) complexes containing two or more Fe(III) centers have been also developed. Their relatively large size and the rigid geometry indeed increases the proton relaxivity by limiting their localized rotational motions. Water soluble metal–organic cages with multiple Fe(III) centers are one of the promising examples. It is also worth noting that activatable iron complexes responding to chemical and physical stimulus such pH, reactive oxygen species, light, have a significant potential to provide molecular contrast agents for MRI. 

In spite of the recent impressive progress of low-molecular-weight Fe(III) complexes summarized above, the design of Fe(III) complexes as alternatives to commercially available Gd(III)-based agents remains in its infancy, and particular attentions are required for the preparation of Fe(III) complexes. (i) The potential reduction of the Fe(III) center in biological environments [74,75] is one of the main concerns and has been often overlooked. The reduction may not only lead to the loss of MRI efficacy in vivo, but also may lead to the formation of harmful radicals such as ·OH, generated directly by Fe(II) species [76]. It has been suggested that the redox potential of the Fe(III)/Fe(II) should be less than 0.1 V or greater than 0.9 V (vs NHE) to prevent redox cyclizing [77]. Otherwise, the iron complexes could be reduced by ascorbate or oxidized by peroxide. (ii) Dissociation of the iron complex under physiological conditions should be also avoided [17,78]. The free ligands may chelate the Ca^2+^ ions, causing disruption of normal function [79]. The dissociated iron ions may activate peroxidase which could lead to the cell apoptosis. Iron overload induced by the free ions could be an additional potential threat, although the human body is able to handle small amounts of excess iron [80]. Thus, the thermodynamic stability constant of the designed iron complex should be large enough, at least bigger than that of the clinically used Gd(III)-based counterparts. (iii) Contrast agents are generally administered in gram quantities to ensure positive and obvious contrast enhancement in the images. This means that the kilo manufacturing of the iron complexes are necessary for their clinical trials. Hence, the preparation of the complexes, particularly the synthesis of the ligands, should be straightforward to lower the investment cost. There is no doubt that low-molecular-weight Fe(III) complexes have a bright future in MRI applications, and may become attractive in diagnosis and therapy.

## Figures and Tables

**Table 2:** Protonation constant (log *K*_1_) and relaxivity (*r*_1_, *r*_2_) for mononuclear Fe(III) complexes with macrocyclic ligands.

Complex	Log *K*_1_^a^	*r*_1 (mM_^−1^_s_^−1^_)_^b^ in Buffer	*r*_2 (mM_^−1^_s_^−1^_)_^b^ in Buffer	*r*_1 (mM_^−1^_s_^−1^_)_^b^ in HSA	*r*_2 (mM_^−1^_s_^−1^_)_^b^ in HSA
Fe(III)–TOB [54]	7.05	2.2	4.5	2.5	4.2
Fe(III)–TOBA [54]	6.75	0.81	1.5	1.1	1.6
Fe(III)–TzB [54]	6.91	1.7	5.0	2.2	3.6
Fe(III)–TzBC [54]	7.44	0.42	1.5	0.96	2.0
Gd(III)–DTPA [54]	22.5 ^c^	3.1	3.9	3.2	4.0
Fe(III)–TOSO [55]	7.55 ^d^	2.0 ^e^	6.1 ^e^	2.5 ^e^	5.5 ^e^
Fe(III)–NOHP [55]	11.78 ^d^	10.97 ^e^	1.8 ^e^	1.2 ^e^	2.3 ^e^
Fe(III)–NOTP [56]	29.6 ^c^	0.72 ^e^	1.26 ^e^	1.05 ^e^	1.55 ^e^
Fe(III)–TOCs [57]	-	1.10	1.6	-	-
Fe(III)–TOCO151 [57]	-	1.06	2.04	-	-
Fe(III)–DTPA [57]	-	0.51	1.20	-	-
Fe(III)–EDTA [57]	-	1.37	2.28	-	-

^a^ Protonation constants from potentiometric titrations at 37 °C, in 0.10 M NaCl over the pH range of 3 to 11. ^b^ Relaxivity values measured at 4.7 T, pH 7.2 and 37 °C. ^c^ Stability constants. ^d^ log *K*_1_ measured by potentiometric titrations at 25 °C, in 0.10 M NaCl, with 1–2 mM meglumine. ^e^ Relaxivity values measured at 4.7 T, pH 7.4 and 37 °C.
